# Clinical Insights and Case Analysis of Disorders Attributed to Cicadas in the Emergency Department

**DOI:** 10.5811/westjem.52962

**Published:** 2026-05-15

**Authors:** Mary Heslin, Jonah Frueh, Madison Watts, Ahmad Abdulla, Alexandras Biskis, Sean M. Fox, Elise Lovell, Ryan McKillip

**Affiliations:** * Advocate Health, Carolinas Medical Center, Department of Emergency Medicine, Charlotte, North Carolina; † Advocate Health, Advocate Christ Medical Center, Department of Emergency Medicine, Oak Lawn, Illinois; ‡ Aurora Sinai Medical Center, Advocate Aurora Research Institute, Office of Research Analytics and Systems Computing, Milwaukee, Wisconsin; § Advocate Health, Advocate Christ Medical Center, Oak Lawn, Illinois; || University of Illinois at Chicago, Chicago, Illinois; # Wake Forest University School of Medicine, Department of Emergency Medicine, Oak Lawn, Illinois

## Abstract

**Introduction:**

In 2024 the United States experienced a rare co-emergence of two periodical cicada broods (XIII and XIX), along with annual cicadas. Although not inherently dangerous, cicadas have been linked to allergic reactions and unintentional injuries. The public health impact of this extraordinary event is poorly understood. In this study we aimed to characterize emergency department (ED) and urgent care (UC) visits associated with the 2024 cicada emergence.

**Methods:**

We conducted a retrospective chart review across ED and UC sites in a large healthcare system in the Midwest and Southeast that coincides with the ranges of the periodical cicada broods, from April 1–July 31, 2024. Electronic health records were searched for “cicada” and common variants. Two emergency physicians in each region reviewed identified records. Data extracted included demographics, diagnoses, visit characteristics, diagnostics, treatments, and outcomes.

**Results:**

Of 1,304,743 total visits, 68 mentioned “cicada” or a variant; 42 were confirmed as cicada related. Patient ages ranged from 7 weeks to 87 years of age (median 38 years). Trauma was the most common cicada-related presentation (33), followed by falls (21), blunt trauma (6), vehicular/bicycle accidents (3), and other mechanisms. Additional cases involved allergic reactions (3), environmental exposure (2), and neurologic symptoms (2). Imaging was common: 57% had radiographs and 43% computed tomography. Seven patients sustained fractures; one required laceration repair, and six were admitted.

**Conclusion:**

While the overall health system impact was limited, cicada-related visits revealed important patterns of injury. Findings support the need for public education and preparedness during future mass insect events.

## INTRODUCTION

Cicadas are winged insects that spend most of their life cycle underground before emerging synchronously in massive numbers to overwhelm predators. These coordinated emergences occur only at specific intervals and within distinct, geographically defined broods found across tropical, subtropical, and temperate regions, including the midwestern and eastern United States.^1^ In 2024 the U.S. experienced the simultaneous emergence of two significant periodical cicada broods, Broods XIX and XIII, an event occurring only every 221 years. Brood XIII emerges every 17 years, and has a range covering northern Illinois, southern Wisconsin, northwestern Indiana, southwestern Michigan, and eastern Iowa. Brood XIX emerges every 13 years, is concentrated in southern Illinois and Missouri, and it is also found across the southeastern states. These are in addition to annually emerging broods. This simultaneous emergence in 2024 resulted in an unprecedented cicada burden of trillions of insects,^2^ leading to a potentially significant public health impact. Our aim in this paper was to identify resource utilization of healthcare services in the emergency department (ED) and urgent care (UC) setting to better understand the impact of this simultaneous cicada emergence.

Research has highlighted various public health and safety concerns posed by cicadas. Insects in general are a common reason for an ED visit. A study analyzing animal-related injuries treated in an ED over two years found that insect-related visits were most common and resulted in a 4.2% hospital admission rate. These were due to allergic reactions or infections because of an insect.^3^

More specific to cicadas, 12 cicada-related cases were reported among children who presented to Cincinnati Children’s Hospital during the 1987 periodical emergence.^4^, ^5^ Injuries included concussions, blunt trauma, a crushed hand, a stab injury, and multiple bicycle accidents. These were all caused by children trying to either kill or avoid cicadas. The extent of traumatic injuries among adults because of cicadas is not well known. An incident in Ohio where a driver crashed his car after a cicada flew through his window reveals the potential for these insects to cause significant accidents and injuries among adults as well.^6^

Ingestion of cicadas poses another health risk, as they have been known to trigger serious allergic reactions when consumed. A notable case report documented a man with a shellfish allergy developing urticaria and throat itching after consuming cicadas, requiring epinephrine and intravenous (IV) steroids. This incident suggests potential cross-reactivity between cicada proteins and shellfish allergens, broadening the scope of cicada-related health risks to include serious allergic reactions.^7^

There is also evidence of cicadas carrying fungi that can be toxic to humans. A study in Thailand identified 39 people who ingested cicadas infected with cordyceps fungus from 2010–2022.^8^ Patients developed gastrointestinal and neurological symptoms, including tremors and seizures, and most required hospitalization. Similar findings were published in 2017 from a study of 60 patients in Southern Vietnam who consumed cordyceps-infected cicadas from 2008–2015.^9^ This fungus is also prevalent throughout the United States.^10^

These studies provide an overview of the predominantly avoidable health risks associated with cicadas, from physical injury to allergic reactions and severe poisoning after cicada consumption. Building on the existing literature, we aimed to characterize ED and UC visits associated with the 2024 cicada emergence to aid in the development of future public health responses to mass insect events.

**Population Health Research Capsule**

What do we already know about this issue?

*Insect-related concerns are a common reason for emergency department (ED) visits, and cicadas have been associated with trauma, allergic reactions, and poisoning*.

What was the research question?

*We characterized ED and urgent care visits associated with the 2024 cicada emergence*.

What was the major finding of the study?

*There was a very modest healthcare system impact. However, individual traumatic morbidity was often substantial*.

How does this improve population health?

*Public health education efforts should focus on the message that cicadas are harmless when left alone*.

## METHODS

We conducted a retrospective chart review of ED and UC visits from April 1–July 31, 2024, the four months of highest cicada emergence concentration, within a large healthcare system with coverage in six affected states: Illinois; Wisconsin; North Carolina; South Carolina; Alabama; and Georgia. The system includes both urban and rural hospitals and UC centers across these regions, allowing a diverse sample of patient encounters.

A comprehensive keyword search was performed in the electronic health record (EHR) for any appearance of the term “cicada” and its common variants, including cicada, cicata, ciccada, sicada, cicaida, cecada, secada, sikada, and sick aid. This query spanned all ED and UC visits during the study period to capture potential charting or dictation errors. Charts were included only if the visit was determined to be directly related to cicada exposure or behavior. Each pertinent patient chart was manually reviewed by two emergency physicians. We compiled extracted information into a de-identified spreadsheet. Discrepancies were resolved by investigator consensus through verbal discussion until a final, agreed upon determination was reached, in accordance with best practices for emergency medicine chart review studies.^11^

Extracted variables included demographics (age, sex), arrival mode (eg, emergency medical services [EMS], walk-in, personal transport), visit type (UC or ED), diagnostic testing (eg, labs, imaging, therapeutics (eg, oral, IV, topical medications), length of stay, and disposition (eg, discharged, admitted). Each visit was assigned to a clinical category determined a priori based on previous literature: trauma; allergic reaction; environmental exposure; or neurologic symptoms. Traumatic presentations were further subclassified by mechanism (eg, fall, blunt trauma, vehicular accident). New categories were created as needed through study team consensus.

We used descriptive statistics for all analyses. Following published methodologic criteria for chart review studies,^12^ we defined case selection criteria and variables, used data abstraction forms, focused on interobserver reliability during investigator meetings, and described the medical record sampling as per above. This study was determined to be exempt from institutional review board (IRB) review by the Wake Forest University School of Medicine IRB under 45 CFR 164.512. The primary outcome of this study was the identification of ED and UC visits related to the 2024 periodical cicada brood emergence. Secondary outcomes included clinical presentation categories, resource utilization, and patient disposition.

## RESULTS

Among 1,304,743 total visits to 118 different EDs and UCs across the system during the study period, 68 mentioned “cicada” or a spelling variant. Of these, 42 were confirmed as cicada related. The age range of affected patients was 7 weeks to 87 years of age (median 38 years). Approximately two-thirds of patients (69%) self-presented, with 31% arriving by EMS. Trauma was the most common visit category (33), including falls (21), blunt trauma (6), vehicular/bicycle incidents (3), muscle strains (2), and one penetrating injury. Additional presentations included allergic reactions (3), environmental exposure (2), neurologic symptoms (2), eye problem (1), and mental health (1) (see Table). Imaging was frequently obtained; 24 patients (57.1%) underwent radiographs and 18 (42.9%) had CT. Seven patients (16.7%) sustained fractures; one required laceration repair. Six patients (14.3%) were admitted. Of the 26 non-related cases, most were excluded due to incidental mention of cicada (“sounds like cicadas in my ears” after cotton swab injury), or non-clinical references (eg, address Cicada Drive).

## DISCUSSION

This study provides the first systematic review of cicada-related health visits during a dual-brood emergence. While the proportion of cicada-related visits was small, individual morbidity was often substantial. Most cases involved trauma, which was largely preventable. Two cases involved passengers leaping from moving vehicles after cicadas flew in through open windows. While other trauma cases were less dramatic, a common theme was patients being injured while attempting to swat, kill, or run away from cicadas. Examples of injuries suffered include a hand laceration suffered while attempting to kill a cicada with a weed whacker; reopening a surgical wound after being swarmed by cicadas at physical therapy; arm injury after falling off a scooter; chest injury from running into a safety pole; and a head injury from running into a wall. While reacting when startled is universal, these injuries suggest an opportunity for public safety messaging about the harmless nature of cicadas.

Although allergic cases were relatively rare, they align with prior literature and support the need to educate about the potential risk of cicada-associated allergic reactions. In addition, a patient with bipolar disorder experienced decompensation, later linked to cicada noise disrupting sleep—underscoring how environmental stimuli can exacerbate mental illness. In other patients, the constant hum of cicadas was described as contributing to headache and lightheadedness. Poisoning from ingesting fungus-infected cicadas can be serious; however, this mechanism of exposure was not seen.^8^

## LIMITATIONS

As a retrospective review, this study depended on accurate documentation and keyword capture. However, the unusual nature of the keyword “cicada” strengthens specificity in our case identification. The oak leaf itch mite, *Pyemotes herfsi*, feeds on cicada eggs and causes severe itching and rash in people exposed to their bites.^13^ Cases involving mite bites may have been missed due to its nonspecific presentation. Selection bias was a potential factor as additional patients with minor complaints may have presented to primary care clinics. However, our goal in this study was to evaluate resource utilization in the ED and UC settings; thus, we intentionally excluded primary care visit data. Some injuries and illness patterns may be regionally specific, but the involved healthcare system is large and includes six states in both the Midwest and Southeast regions of the country.

## CONCLUSION

The 2024 emergence of cicada Broods XIII and XIX led to modest but meaningful cicada-related health visits. Most were associated with trauma and preventable. This study highlights the need for targeted public health messaging to mitigate injury during future cicada events, especially through awareness campaigns that cicadas are harmless when left alone.

## Figures and Tables

**Table:** Patient presentations to a large health system in a retrospective study of diagnoses and outcomes related to emergence of cicada insect broods in 2024.

Patient presentation by clinical category	n (%)
Trauma	33 (78.6)
Allergic reactions	3 (7.1)
Environmental exposure	2 (4.8)
Neurologic symptoms	2 (4.8)
Eye problem	1 (2.4)
Mental health	1 (2.4)
